# Mortality from chronic respiratory disease in Brazil: time trend and forecasts

**DOI:** 10.11606/s1518-8787.2022056003672

**Published:** 2022-06-07

**Authors:** Marcio Sacramento de Oliveira, Elisa Hypólito Montovani, Maria de Fátima Ebole de Santana, Antonio Carlos Monteiro Ponce de Leon, Márcio Candeias Marques

**Affiliations:** I Fundação Oswaldo Cruz Instituto de Comunicação e Informação Científica e Tecnológica em Saúde Rio de Janeiro RJ Brasil Fundação Oswaldo Cruz. Instituto de Comunicação e Informação Científica e Tecnológica em Saúde. Rio de Janeiro, RJ, Brasil; II Universidade do Estado do Rio de Janeiro Instituto de Medicina Social Rio de Janeiro RJ Brasil Universidade do Estado do Rio de Janeiro. Instituto de Medicina Social. Rio de Janeiro, RJ, Brasil; III Fundação Oswaldo Cruz Escola Politécnica de Saúde Joaquim Venâncio Rio de Janeiro RJ Brasil Fundação Oswaldo Cruz. Escola Politécnica de Saúde Joaquim Venâncio. Rio de Janeiro, RJ, Brasil

**Keywords:** Respiratory Tract Diseases, mortality, Chronic Disease Indicators, Health Programs and Plans, trends, Ecological Studies

## Abstract

**OBJECTIVE:**

To analyze the time trend of monthly mortality rates from chronic respiratory diseases in Brazil from 1996 to 2017, with forecasts for 2022, besides analyzing the possibility of achieving the goal of the *Plano de Ações Estratégicas para o Enfrentamento das Doenças Crônicas Não Transmissíveis no Brasil* (Strategic Action Plan to Tackle Chronic Noncommunicable Diseases in Brazil) from 2011 to 2022.

**METHODS:**

This is an ecological study that uses data from *Sistema de Informações sobre Mortalidade* (SIM – Mortality Information System), S*istema de Informações Demográficas e Socioeconômicas* (Demographic and Socioeconomic Information System) and *Pesquisa Nacional por Amostra de Domicílios Contínua* (PNAD Contínua – Continuous National Household Sample Survey). We established the age range between 30 and 69 years old and the evolution of the rates over time was made by autoregressive integrated moving average models in R statistical tool.

**RESULTS:**

Premature mortality rates from chronic respiratory diseases are decreasing in Brazil as a whole, mostly in state capitals. There is also a trend to reach the Ministry of Health’s goal in most of the country. For capitals that tend not to reach the goal, there is an association between mortality and social indicators, healthcare network and frequency of smoking.

**CONCLUSION:**

This study intends to improve planning of the public health system for the control of chronic respiratory diseases.

## INTRODUCTION

The worldwide premature mortality rate from chronic noncommunicable diseases (NCDs) dropped by 18% at the beginning of this century^1^. However, this progress was not sustained since the decline of mortality from NCDs slowed down^1^. These diseases, mainly cardiovascular diseases, cancers, chronic respiratory diseases and diabetes, correspond to the largest number of deaths in the world (63% annually) and, once demographic changes compensate for the drop in mortality rates, NCDs are taking an even higher proportion of total deaths^1,2^.

In the Americas, the percentage reduction rate of premature mortality from NCDs also decreased^3^. Despite leading as the cause of morbidity and mortality in Brazil, there was a 20% decline in the mortality rate from NCD between 1996 and 2007^4^, and a reduction in the premature mortality rate (30 to 69 years old) from NCDs, from 2000 to 2013 (2.5% per year), with chronic respiratory diseases (CRD) leading the percentage variation (4.1%)^5^.

The CRD alone was the third cause of death (7%) in the world in 2017, after cardiovascular and neoplastic diseases, especially chronic obstructive pulmonary disease (COPD) and asthma^6^. Between 1990 and 2017, the total number of deaths caused by CRD increased worldwide, whereas the age-standardized mortality rate decreased^6,7^. The CRD scenario in Brazil followed the global trend, with COPD being the fourth cause of death in 2019, with an absolute increase in the number of deaths from CRD and a reduction in age-adjusted mortality rates^5,8^.

The NCDs mainly affect low-income and low-education populations, as they are more exposed to risk factors and have reduced access to health services^4,11^. In the case of CRD, it is not different. Smoking is the main risk factor associated with years of life adjusted for disability^8^. In this context, studies show that less socioeconomically favored groups are more prone to use tobacco frequently^12^. Moreover, data from *Vigilância de Fatores de Risco e Proteção para Doenças Crônicas por Inquérito Telefônico* (Vigitel – Surveillance System for Risk and Protective Factors for Chronic Diseases by Telephone Survey)^13^ indicate lower frequency of smoking with higher schooling. Furthermore, the poorest are less diagnosed with CRD and those who use fewer medications^14^.

Facing the impact of NCDs, global and national policies are committed to addressing these diseases. The first High-Level Meeting on the Prevention and Control of NCDs in 2011; target 3.4 from Sustainable Development Goals (SDG), of reducing by one-third premature mortality from these diseases by 2030; and the Global Action Plan for the Prevention and Control of Noncommunicable Diseases 2013–2020 were highlights on the world agenda^2,15^.

In Brazil, the implementation of the Surveillance System for NCDs was relevant and allowed structuring actions to monitor risk factors, morbidity and mortality of NCDs^4,16^. The Ministry of Health (MS) also built the *Plano de Ações Estratégicas para o Enfrentamento das Doenças Crônicas Não Transmissíveis* (Strategic Action Plan to Tackle NCDs) in Brazil, 2011–2022, having as one of the goals reducing the premature mortality rate from NCDs by 2% per year^4^.

The country has been considered an example in the fight against smoking, recognized by world organizations, with emphasis on the increase in the tax on cigarettes and the increase in warnings on packaging, by Law No. 12,546 of 2011^16^.

However, globally, CRD received fewer resources for research and less public attention than other diseases, such as cardiovascular and neoplastic ones^17^. The present study intends to analyze the time trend of mortality from CRD in Brazil, forecasting rates for 2022, besides analyzing, for each state capital, the possibility of achieving the goal of the Strategic Action Plan to Tackle NCDs in Brazil, 2011–2022.

## METHODS

This is a time-series ecological study of monthly mortality rates from CRD (ICD-10: J30–J98) between 1996 and 2017, with forecast for 2022, in Brazilian capitals and in Brazil as a whole. Data on deaths were obtained from the Brazilian Unified Health System Computer Department (Datasus) Mortality Information System – Ministry of Health (SIM/MS), and population data from 1996 to 2015 were acquired from the Demographic and Socioeconomic Information System, also from Datasus/MS. The estimated total population for 2016 and 2017 was made based on the continuous *Pesquisa Nacional por Amostra de Domicílios* (PNAD – National Household Sample Survey), using weighed samples to calculate the total population.

The monthly general crude mortality rates from CRD and monthly age-standardized rates were calculated using the method of direct standardization, which enabled the comparison of the results of the different populations by age group. The method selects a standard population and applies the mortality rate of all units to that same population. The Brazilian population in 2010 (census year) was used; mortality rates were calculated and represented by 100,000 inhabitants.

The chosen age range was 30 to 69 years and allowed us to compare the time trend of mortality from CRD in Brazil and the goal proposed by the Strategic Action Plan to Tackle NCDs.

Descriptive data analysis was used to explore the behavior of monthly mortality rates from CRD, obtaining values of minimum, first quartile, median, mean, third quartile, maximum and standard deviation. Based on the mean and standard deviation, one can calculate the coefficient of variation of the rates, which is important to express the dispersion of the data and compare values without the influence of the differences in magnitude of the variable.

The evolution of mortality rates in relation to time (months/years) was made by autoregressive integrated moving average (ARIMA) models. The time-series models of this type allow the dependent variable, mortality from CRD, to be represented by Y_t_ and explained by lagged values (Y_t-n_), from Y_t_ itself, different from the regression models, in which Y_t_ is explained by the regressors μ, X1, X2, X3, ... Xn, without influence of time^18^. Thus, μ is the constant of the model and X1, X2, X3, ... Xn are explanatory variables. To complement ARIMA models, the model with autoregressive distributed lag (dynamic regression) was also used. The variables used were the standardized mortality rate, the time and this same rate delayed in time (t-1).

Assuming that the mortality rate follows a normal distribution (Gaussian) and that it is a realization of a stationary stochastic process, we applied the dynamic regression for each capital, as well as for Brazil. The lag order for each capital and Brazil (t-1) was determined by the autocorrelation function. Being the model in general given by:


$$y_{t}\;\;=\;\alpha \;+\;\beta x_{t}\;\;+\;\gamma_{1}\;y_{t-1}+\;\gamma_{2}\;y_{t-2}+\;\gamma_{3}\;y_{t-3}+\;\theta_{1}\;u_{t-1}+\;\theta_{2}\;u_{t-2}+\;\theta_{3}\;u_{t-3}+\;\Theta_{1}\;u_{t-12}+\;\Gamma_{1}\;y_{t-12}+\;u_{t}$$

Where *Y*_t_ is the value of the dependent variable at instant *t*; γ is the parameter attached to the autoregressive operator; θ is the parameter attached to the moving average operator; and Θ and Γ are seasonal factors. For ARIMA models, the first difference was denoted as $∆z_{t}\;\;=\;{z_{t}}_{\;}–\;z_{t-1}$ being *Y*_*t*_ = ∆*z*_*t*_ for the purpose of writing the estimated models. The components trend, seasonality, cycle and error were decomposed by the X12-ARIMA methodology and the trend evaluated as increasing or decreasing. The Dickey-Fuller test increased was performed for all series, which presented unit root, and, for this reason, the first difference was used. Autocorrelation functions also presented a slow decay.

The model was estimated by the function of maximum likelihood. For the modeling process, the first three years of Rio de Janeiro were suppressed.

Regarding 19 capitals (Belém, Boa Vista, Macapá, Manaus, Palmas, Porto Velho, Rio Branco, João Pessoa, Maceió, Salvador, São Luís, Teresina, Brasília, Campo Grande, Cuiabá, Goiânia, Vitória, Curitiba and Florianópolis), the outliers had to be replaced with the mean mortality rates of their respective years to achieve better adequacy of the models.

The evaluation of the overall performance of the model was based on the prediction of monthly mortality rates from CRD for 2017 and the comparison with the actual values of that year, which made it possible to calculate the percentage of the mean absolute percentage error (MAPE). In this procedure, the rates for 2017 were deleted from the model and estimated in the remainder. The maximum MAPE value for the model to be considered valid for forecasting was defined as 50%. After the validating the model, forecast until December 2022 was made.

The predicted percentage change for the mortality rate was calculated based on the value of the 2011 standardized annual rate, the year of preparation of the Ministry of Health plan, and the expected mortality rate for 2022. The result of each Brazilian capital was compared, as well as the one of all Brazil, with the document proposal (a 19.93% drop in the premature mortality rate until 2022, value obtained from the calculation of the accumulated rate of 2% per year), evaluating which units show a trend or not to reach the target.

Data were analyzed in R statistical tool^19^, version 3.6.3, using the Forecast^20^and Imtest^21^packages.

## RESULTS

From the analysis of minimum and maximum values from mortality rates, presented in Table 1, we observed the lowest monthly rates in Boa Vista, Palmas, Porto Velho, Rio Branco, Aracaju, João Pessoa, São Luís, Teresina, Campo Grande, Vitória and Florianópolis; the highest value was found in Boa Vista (15.04%).

**Table 1:** Descriptive analysis of monthly mortality rates from chronic respiratory diseases, per 100,000 inhabitants, in Brazilian capitals and Brazil as a whole, 1996–2017.

Location	Min.	Q1	Median	Mean	Q3	Max.	SD
Brasil	1.32	1.88	2.19	2.33	2.72	4.59	0.59
Belém	0.60	2.02	2.72	3.05	3.82	8.38	1.44
Boa Vista	0.00	0.00	1.34	1.87	2.71	15.04	2.33
Macapá	0.26	0.99	1.50	1.59	2.02	5.32	0.78
Manaus	0.31	1.23	1.83	1.92	2.46	4.82	0.88
Palmas	0.00	0.00	0.93	1.74	2.85	20.09	2.60
Porto Velho	0.00	1.43	2.66	3.18	4.23	12.54	2.40
Rio Branco	0.00	1.67	3.22	3.40	4.87	12.71	2.28
Aracaju	0.00	1.08	1.92	2.33	3.42	7.83	1.56
Fortaleza	0.44	1.46	2.10	2.33	3.04	5.66	1.06
João Pessoa	0.00	1.32	1.92	2.14	2.80	7.29	1.20
Maceió	0.35	1.61	2.45	2.78	3.64	8.61	1.50
Natal	0.00	0.79	1.21	1.33	1.71	4.25	0.80
Recife	1.21	2.34	2.88	2.99	3.57	6.22	0.90
Salvador	0.72	2.24	3.41	3.54	4.63	8.94	1.56
São Luís	0.00	1.19	1.77	1.97	2.50	5.72	1.08
Teresina	0.00	1.07	1.70	1.88	2.50	7.08	1.14
Brasília	0.26	0.99	1.50	1.59	2.02	5.32	0.78
Campo Grande	0.00	1.33	2.02	2.10	2.74	6.12	1.05
Cuiabá	0.00	1.44	2.38	2.59	3.39	9.43	1.55
Goiânia	0.58	1.79	2.46	2.61	3.19	6.63	1.14
Belo Horizonte	0.52	1.18	1.68	1.92	2.46	6.25	0.97
Rio de Janeiro	1.20	1.83	2.24	2.42	2.88	5.15	0.78
São Paulo	1.13	1.70	2.12	2.25	2.73	5.07	0.70
Vitória	0.00	0.56	1.14	1.29	1.90	3.94	0.97
Curitiba	0.35	1.26	1.93	2.23	2.93	7.88	1.31
Florianópolis	0.00	0.93	1.62	1.92	2.59	7.09	1.32
Porto Alegre	0.93	2.08	2.73	3.06	3.88	9.90	1.43

Q1: first quartile; Q3: third quartile; SD: standard deviation.

The comparison between the mean and median data in Table 1, for the mortality rates of each capital, showed that Palmas has the highest number of outliers.

We observed a significant variation in the monthly mortality rates from CRD in all units studied, which can be noted from their mean and standard deviation values presented in Table 1. All capitals, and Brazil as a whole, presented a coefficient of variation higher than 25%, with the lowest value calculated for the country (25.14%). In turn, Boa Vista and Palmas showed coefficients of variation higher than 100% (respectively, 124.41% and 149.49%).

Regarding the accuracy of the forecasts, we observed in the MAPE, in Table 2, that the models of Brazil (2.94%), São Paulo (12.93%) and Rio de Janeiro (13.81%) have the lowest average percentage error in relation to the others. The models of Boa Vista, Macapá, Manaus, Aracaju, João Pessoa, Campo Grande and Vitória presented high error values, showing a low-accurate forecasting capacity. The error values of the capitals Porto Velho, Rio Branco, Natal, Teresina and Cuiabá show that their models are not suitable for forecasting. Cuiabá, besides the medium-high percentage error, showed a model with behavior in levels, making it difficult to analyze the trend.

**Table 2:** Predictive model, performance and trend of monthly mortality rates from chronic respiratory diseases, per 100,000 inhabitants, from Brazilian capitals and Brazil as a whole, 1996–2017.

Location	Equation	Sigma2	MAPE (%)	Trend
Brasil	∆*ẑ*_*t*_ = 0,5785∆*z*_*t*–1_ – 0,9253*u*_*t*–1_ – 0,4879*u*_*t*–12_ –0,2251*u*_*t*–13_	0.02	2.94	Decreasing
Belém	∆*ẑ*_*t*_ = –0,4177∆*z*_*t*–1_ – 0,3695*u*_*t*–1_ – 0,4865*u*_*t*–2_ – 0,8696∆*z*_*t*–12_ + 0,0281*u*_*t*–1*2*_ – 0,7212*u*_*t*–13_ – 0,0964*u*_*t*–14_ – 0,2105*u*_*t*–15_	1.06	29.16	Increasing
Boa Vista	∆*ẑ*_*t*_ = –0,8365∆*z*_*t*–1_ – 0,1131*u*_*t*–1_ – 0,8126*u*_*t*–2_ + 0,2129∆*z*_*t*–12_ – 0,1860*u*_*t*–12_ – 0,0713*u*_*t*–2_	2.34	39.13	Decreasing
Macapá	∆*ẑ*_*t*_ = –0,1152∆*z*_*t*–1_ –0,9375*u*_*t*–1_ –0,610∆*z*_*t*–12_ + 0,6822*u*_*t*–12_ + 0,1924*u*_*t*–13_ –0,0045*x*_*t*_	1.79	41.33	Decreasing
Manaus	∆*ẑ*_*t*_ = –0,0755∆*z*_*t*–1_–0,9305*u*_*t*–1_ + 0,8866∆*z*_*t*–12_ –0,8506*u*_*t*–12_ + 0,1254*u*_*t*–13_ –0,0042*x*_*t*_	0.58	44.37	Decreasing
Palmas	∆*ẑ*_*t*_ = –0,6530∆*z*_*t*–1_ – 0,2855*u*_*t*–1_ – 0,6947*u*_*t*–2_ + 0,1092*u*_*t*–3_ – 0,1290*u*_*t*–4_ – 0,4751∆*z*_*t*–12_	3.69	29.42	Increasing
Porto Velho	∆*ẑ*_*t*_ = –0,8657*u*_*t*–1_–0,1249*u*_*t*–12_	2.86	69.79	NA
Rio Branco	∆*ẑ*_*t*_ = –0,7347∆*z*_*t*–1_ – 0,5022∆*z*_*t*–2_ – 0,3746∆*z*_*t*–3_ – 0,2346∆*z*_*t*–4_	5.23	83.79	NA
Aracaju	∆*ẑ*_*t*_ = –0,0973∆*z*_*t*–1_ + 0,0956∆*z*_*t*–2_ – 0,2061∆*z*_*t*–3_ – 0,8573*u*_*t*–1_	1.52	40.99	Decreasing
Fortaleza	∆*ẑ*_*t*_ = 0,2635∆*z*_*t*–1_ – 0,9302*u*_*t*–1_ – 0,0085*x*_*t*_	0.48	28.87	Decreasing
João Pessoa	∆*ẑ*_*t*_ = –0,7849∆*z*_*t*–1_ – 0,0480*u*_*t*–1_ – 0,7135*u*_*t*–2_ – 0,0083*x*_*t*_	0.92	49.62	Decreasing
Maceió	∆*ẑ*_*t*_ = –0,8756∆*z*_*t*–1_ + 0,0225∆*z*_*t*–12_ – 0,0163*u*_*t*–12_ – 0,0104*x*_*t*_	1.16	34.27	Decreasing
Natal	∆*ẑ*_*t*_ = –0,8459*u*_*t*–1_	0.42	91.66	NA
Recife	∆*ẑ*_*t*_ = –0,8636*u*_*t*–1_ – 0,7568∆*z*_*t*–12_ – 0,0654∆*z*_*t*–13_ + 0,7071*u*_*t*–12_	0.76	22.81	Increasing
Salvador	∆*ẑ*_*t*_ = 0,2305∆*z*_*t*–1_ – 0,9429*u*_*t*–1_ – 0,0161*x*_*t*_	0.60	28.55	Decreasing
São Luís	∆*ẑ*_*t*_ = 0,1169∆*z*_*t*–1_ – 0,6843∆*z*_*t*–2_ – 0,9558*u*_*t*–1_ + 0,5828*u*_*t*–2_ – 0,4866*u*_*t*–3_ – 0,0225*u*_*t*–4_ – 0,0824*u*_*t*–5_	0.63	35.33	Increasing
Teresina	∆*ẑ*_*t*_ = 0,1373∆*z*_*t*–1_ – 0,9492*u*_*t*–1_ + 0,1342∆*z*_*t*–12_ – 0,0171∆*z*_*t*–13_	0.70	104.75	NA
Brasília	∆*ẑ*_*t*_ = –0,1638∆*z*_*t*–1_ – 0,8374∆*z*_*t*–2_ – 0,0663∆*z*_*t*–3_ – 0,4886∆*z*_*t*–4_ – 0,6258*u*_*t*–1_ + 0,9044*u*_*t*–2_ – 0,9111*u*_*t*–3_ + 0,4809*u*_*t*–4_ – 0,7722*u*_*t*–5_ + 0,0348∆*z*_*t*–12_ + 0,1801∆*z*_*t*–13_ – 0,0076*x*_*t*_	0.27	41.60	Decreasing
Campo Grande	∆*ẑ*_*t*_ = –0,7989∆*z*_*t*–1_ + 0,1317∆*z*_*t*–2_ + 0,1151∆*z*_*t*–3_–0,1016*u*_*t*–1_–0,8287*u*_*t*–2_ + 0,0206∆*z*_*t*–12_ + 0,0883∆*z*_*t*–13_	0.78	33.33	Increasing
Cuiabá	∆*ẑ*_*t*_ = –0,9197*u*_*t*–1_	1.40	162.21	NA
Goiânia	∆*ẑ*_*t*_ = 0,0376∆*z*_*t*–1_ + 0,1849∆*z*_*t*–2_ – 0,8867*u*_*t*–1_ – 0,6050∆*z*_*t*–12_ – 0,2614∆*z*_*t*–13_	0.86	33.58	Decreasing
Belo Horizonte	∆*ẑ*_*t*_ = –0,6022∆*z*_*t*–1_ – 0,8742∆*z*_*t*–2_ – 0,2019*u*_*t*–1_ + 0,3535*u*_*t*–2_ – 0,7581*u*_*t*–3_ – 0,2362*u*_*t*–4_ + 0,2448∆*z*_*t*–12_ – 0,010*x*_*t*_	0.28	25.10	Decreasing
Rio de Janeiro	∆*ẑ*_*t*_ = 0,3222∆*z*_*t*–1_ + 0,1314∆*z*_*t*–2_ – 0,9127*u*_*t*–1_ + 0,1836∆*z*_*t*–12_ – 0,0110*x*_*t*_	0.15	13.81	Decreasing
São Paulo	∆*ẑ*_*t*_ = 0,3580∆*z*_*t*–1_ – 0,9188*u*_*t*–1_ + 0,2552*u*_*t*–12_ + 0,2628*u*_*t*–13_ – 0,0071*x*_*t*_	0.12	12.93	Decreasing
Vitória	∆*ẑ*_*t*_ = –0,7866∆*z*_*t*–1_ – 0,1029∆*z*_*t*–2_ – 0,1812*u*_*t*–1_ – 0,7338*u*_*t*–2_ + 0,4307∆*z*_*t*–12_ – 0,4943*u*_*t*–12_	0.91	47.10	Increasing
Curitiba	∆*ẑ*_*t*_ = 0,02542∆*z*_*t*–1_ – 0,9633*u*_*t*–1_ – 0,7916∆*z*_*t*–12_ + 0,139*u*_*t*–12_ – 0,5454*u*_*t*–13_	0.52	35.63	Increasing
Florianópolis	∆*ẑ*_*t*_ = –0,9353*u*_*t*–1_ – 0,8766*u*_*t*–12_	1.10	37.01	Decreasing
Porto Alegre	∆*ẑ*_*t*_ = 0,0204∆*z*_*t*–1_ – 0,7605∆*z*_*t*–2_ – 0,6562*u*_*t*–1_ + 0,6890*u*_*t*–2_ – 0,7344*u*_*t*–3_ – 0,1944*u*_*t*–4_ + 0,3525∆*z*_*t*–12_ + 0,2395∆*z*_*t*–13_	0.80	34.00	Decreasing

NA: not applicable; MAPE: mean absolute percentage error.

**Figure 1:**
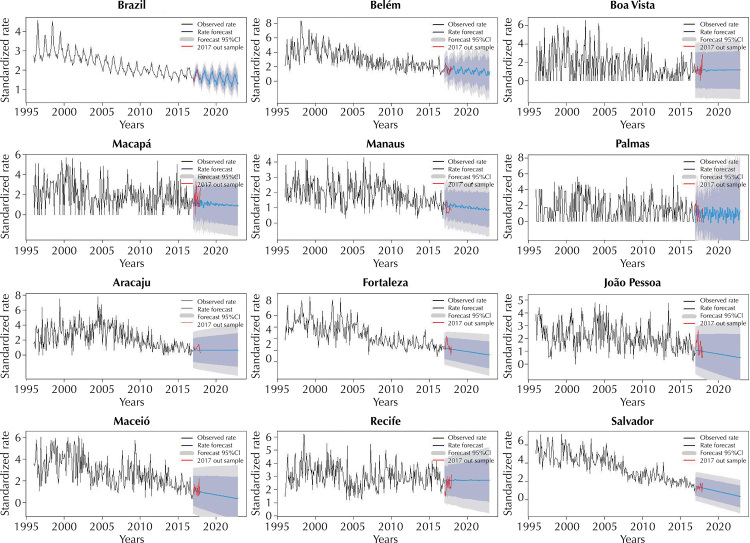
Monthly mortality rates from chronic respiratory diseases adjusted for Brazil as a whole, Belém, Boa Vista, Macapá, Manaus, Palmas, Aracaju, Fortaleza, João Pessoa, Maceió, Recife and Salvador – from 1996 to 2016, with 2017 for validation and forecast of monthly mortality rates from CRD from 01/01/2017 to 12/12/2022 – Forecast by ARIMA model.

CRD: chronic respiratory diseases; ARIMA: autoregressive integrated moving average.

**Figure 2:**
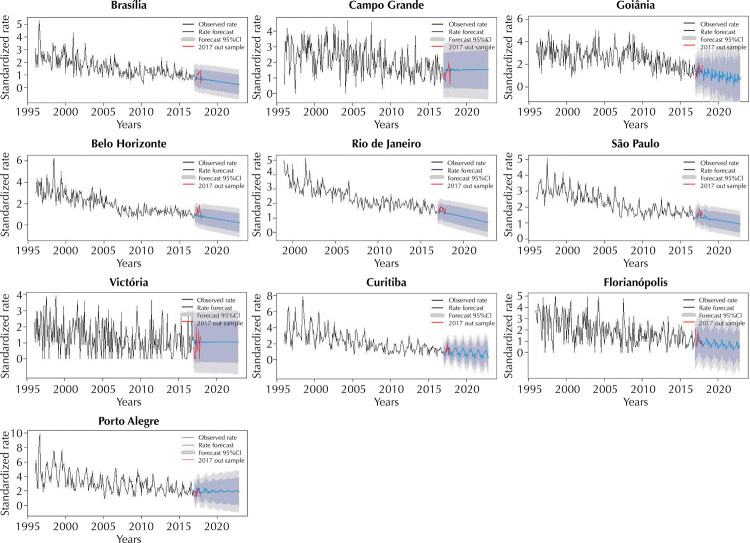
Monthly mortality rates from chronic respiratory diseases adjusted for Brasília, Campo Grande, Goiânia, Belo Horizonte, Rio de Janeiro, São Paulo, Vitória, Curitiba, Florianópolis and Porto Alegre – from 1996 to 2016, with 2017 for validation and forecast of monthly mortality rates from CRD from 01/01/2017 to 12/12/2022 – Forecast by ARIMA model.

CRD: chronic respiratory diseases; ARIMA: autoregressive integrated moving average.

Based on Table 2 and Figures 1 and 2, we note that premature mortality rates are decreasing in Brazil and in 15 of the 22 capitals that had their models validated. Belém, Palmas, Recife, São Luís, Campo Grande, Vitória and Curitiba, on the other hand, show these rates in an increasing trend. For Rio de Janeiro, mortality from CRD data between 1996 and 1998 were much lower than the others, indicating a probable underreporting during this period and, therefore, they were disregarded.

According to Table 3, we also verified that Brazil as a whole and 16 of the 22 capitals tend to reach the goal of the Strategic Action Plan to Tackle NCDs, as they present a higher-than-expected predicted percentage variation for the premature mortality rates from CRD (19.92%). For Boa Vista, Recife, São Luís, Campo Grande and Vitória, the results show a trend not to reach the goal. The capital Boa Vista showed the lowest rate in 2011 (10.04), whereas the highest was seen in Rio Branco (36.76).

**Table 3:** Mortality rates from chronic respiratory diseases, per 100,000 inhabitants, expected for 2022, and evaluation of the achievement of the goal of the Strategic Action Plan to Tackle NCDs, for Brazil and Brazilian capitals.

Location	Standardized rate, 2011	Standardized rate, 2022 (National Plan)	Standardized rate, 2022 (expected)	Expected variation % (2011–2022)	National Plan’s Goal
Brazil	23.65	18.94	17.45	-26.22	Tends to reach
Belém	23.11	18.51	14.28	-38.19	Tends to reach
Boa Vista	10.04	8.04	13.63	+35.80	Tends not to reach
Macapá	14.85	11.89	11.03	-25.69	Tends to reach
Manaus	20.51	16.42	10.69	-47.86	Tends to reach
Palmas	18.69	14.97	10.28	-44.98	Tends to reach
Porto Velho	30.51	24.43	NA	NA	NA
Rio Branco	36.76	29.43	NA	NA	NA
Aracaju	18.40	14.74	7.94	-56.87	Tends to reach
Fortaleza	19.23	15.40	7.28	-62.14	Tends to reach
João Pessoa	21.74	17.41	6.22	-71.39	Tends to reach
Maceió	28.71	22.99	4.78	-83.35	Tends to reach
Natal	13.35	10.69	NA	NA	NA
Recife	35.64	28.54	32.43	-9.01	Tends not to reach
Salvador	33.30	26.66	4.86	-84.41	Tends to reach
São Luís	16.28	13.04	17.01	+4.50	Tends not to reach
Teresina	20.37	16.31	NA	NA	NA
Brasília	13.94	11.16	3.11	-77.70	Tends to reach
Campo Grande	19.92	15.95	18.11	-9.13	Tends not to reach
Cuiabá	21.84	17.49	NA	NA	NA
Goiânia	22.96	18.38	8.30	-63.84	Tends to reach
Belo Horizonte	15.72	12.59	2.72	-82.67	Tends to reach
Rio de Janeiro	24.58	19.68	8.94	-63.61	Tends to reach
São Paulo	22.47	17.99	11.55	-48.62	Tends to reach
Vitória	15.11	12.10	12.41	-17.86	Tends not to reach
Curitiba	16.57	13.27	7.79	-52.97	Tends to reach
Florianópolis	17.56	14.06	6.28	-64.20	Tends to reach
Porto Alegre	30.89	24.73	23.25	-24.73	Tends to reach

NCDs: chronic noncommunicable diseases; NA: not applicable.

## DISCUSSION

The downward trend in premature mortality rates from CRD in most of Brazil coincides with results of global research, which show the largest reductions in the burden of CRD in Latin America, and other Brazilian studies that indicate a decrease in those rates in the five regions of the country^5,6,8^. This decline has been attributed, among other factors, to advances in the fight against smoking^5^. Actions in this sense are related to *Eixo II* – *Promoção da Saúde – do Plano de Ações Estratégicas para o enfrentamento das DCNT* (Axis II – Health Promotion – of the Strategic Action Plan to Tackle NCDs) in Brazil. The increase in the access and use of health services also seems to corroborate, according to a study that assessed data from the 2013 *Pesquisa Nacional de Saúde* (PNS – National Health Survey)^22^.

According to the *Síntese de Indicadores Sociais* (SIS – Summary of Social Indicators), from the *Instituto Brasileiro de Geografia e Estatística* (IBGE – Brazilian Institute of Geography and Statistics), there was a drop in the proportion of people living below the poverty line between 2012 and 2014, an important social determinant in the health-disease process, but it grew again since 2015^23^. Another significant determinant is schooling, which presented an increase in the level of education over the last decades that may be related to the reduction of exposure to CRD risk factors, although this higher schooling occurred unequally among different socioeconomic classes^23^. In this context, there seems to be a relation between asthma mortality and factors such as schooling and income distribution, explaining the low mortality from this disease in high-income countries^24^. For COPD, this association is less monotonic and results from increased air pollution and smoking in countries in demographic and epidemiological transition, a causality that deserves specific research in a future study^24^.

Knowing that the highest mortality rates from CRD were found in South Asia, a study that analyzed the trend of prevalence, lethality and risk factors for CRD in India allows comparing the evolution of tackling these chronic diseases in another developing country to the situation in Brazil^6,25^. India’s 2017 National Health Policy recommends reducing mortality from NCDs, including CRD, by 25% by 2025, whereas the study showed decreases greater than 30% in crude mortality rates from COPD and asthma between 1990 and 2016^25^.

The trend of reaching the goal in most of Brazil corroborates a study by Malta (2016), who described the advances in MS’s plan after five years of implementation, in which monitoring by Vigitel demonstrated a reduction in premature mortality from the four main groups of NCDs, and a decrease in tobacco consumption^26^. The study highlights other actions in surveillance such as the performance of the PNS in 2013 and the *Pesquisa Nacional de Saúde do Escolar* (PeNSE – National Survey on Students’ Health) since 2009; besides initiatives in health and comprehensive care promotion, such as the redefinition of the *Rede de Atenção à Pessoa com Doença Crônica* (Care for People with Chronic Diseases Network), the release of the *Programa Nacional de Melhoria do Acesso e da Qualidade da Atenção Básica* (PMAQ – National Program for Access and Quality Improvement in Primary Care) and the *Programa Farmácia Popular do Brasil* (Brazilian Popular Pharmacy Program)^26^. The partnerships with educational and research institutions in structuring NCDs surveillance and funding, in 2011, the implementation of MS’s plan are also notewhorthy^16^.

To better interpret the results of the trend to reach or not the goal, we investigated some factors: health care situation; social indicators; and exposure to smoking.

We studied the situation of health care by the *Cadastro Nacional de Estabelecimentos de Saúde* (CNES – National Registry of Health Facilities) and an article that estimated access to drug treatment of CRD^14,26^. São Luís and Boa Vista, capitals that tend not to reach the goal, have a high relationship between population number and number of health facilities, the first one with 1,056.15 inhabitants for each establishment and the second one with 1,055.17 people per unit, what demonstrates possible health system overload in these municipalities^26^. However, considering only public establishments, the ratio between population and number of establishments ranks Boa Vista as the capital with the second-best relation, showing less overload of the public health system. Therefore, although this ratio is high, 3,485.26 inhabitants per public establishment, alone it does not justify the trend of the municipality not to reach the goal of MS’s plan, demonstrating probable influence of other factors.

Another capital that tends not to reach the goal is Recife, which has a ratio of 720.19 inhabitants per health facility, the 12th worst ratio, improving six positions when the restriction to public units is made. Vitória and Campo Grande have worse overload placements when disregarded private establishments, the first one presenting the best relation between all capitals when considered public and private establishments. Thus, the private sphere would be responsible for reducing the health system overload in these capitals, which could not possibly solve the issue of CRD, once most people depend on the Brazilian Unified Health System (SUS). Thus, it is possible that there is a relation between the ratio between population and number of health facilities and the time trend of mortality from CRD, which is best seen for São Luís and Boa Vista, but this influence is unclear and cannot be analyzed without considering other factors.

About access to medicines for CRD, Leal et al.^14^ (2018) observed that, for individuals who claimed to have medical indication for pharmacological treatment, the prevalence of the use of medicines was lower among residents from North of Brazil (84%), followed by Northeast (92.8%)^14^. As two of the five capitals that tend not to reach the reduction target belong to the northeast region, and one to the north region, there may be a relation between this trend and low access to drug treatment, which is not valid for Campo Grande, because Midwest obtained the best prevalence (98%) of access to medicines for CRD. Southeast region of Brazil presented an 89.7% prevalence, ranking third in the country.

Regarding social indicators, on the one hand, São Luís is the second capital with the lowest mean monthly household income *per capita* (R$ 1,043.00), higher only than Macapá’s income. On the other hand, Vitória, which also tends not to reach the goal, has the highest mean income (R$ 2,988.00) among all capitals^22^. About concentration of wealth, we observed, from the Gini index, a classic inequality index that shows the distribution of *per capita* household income, in which Recife stands out for the greater inequality in income distribution^27^.

São Luís and Recife, as well as Boa Vista, also present a high proportion of people below the poverty line, which corroborates the idea that social indicators influence the trend of mortality from CRD in these capitals^23^. The issue of schooling, an index in which Recife and São Luís showed lower rates than most capitals (98.80% and 98.50%, respectively),^28^ among people aged between six and 14 years old, is also included. In an opposite way, Vitória and Campo Grande showed 100% schooling for this age group^28^.

The frequency of adults who smoke is the highest in Porto Alegre (14.40%) and lowest in São Luís (4.80%)^13^; Campo Grande also has a high rate in relation to other capitals (10.80%), whereas the frequency in Boa Vista represents the median (7.20%), the same value observed in Recife and close to the one from Vitória (7.60%)^13^. The distribution is similar for the percentage of adults who smoke 20 or more cigarettes per day, with Porto Alegre leading, the frequency of São Luís being higher only than the one from Macapá^13^. Therefore, the association between the frequency of this risk factor and mortality from CRD is better seen in Campo Grande.

We based the choice of the SIM as the source of death data on studies that show its adequate performance, being well evaluated in usability, reliability, safety and quality in use^29^. SIM is MS’s oldest information system, implemented in 1976 and, since then, expanding its stability and geographic coverage^29,30^. However, studies indicate limitations, mostly in interoperability and, especially in the North and Northeast, high under-registration and number of deaths from undefined causes^29,30^. Another limitation of the study is the generation of large confidence intervals of ARIMA models, for the forecast for 2022, even with a reasonable predictive capacity with 32.21% average percentage error for 2017, year left out of the estimate in order to test the forecasts.

## CONCLUSIONS

The study demonstrates that early mortality rates from CRD are decreasing in Brazil as a whole and in most capitals, and we can observe in most of the country the trend to achieve the goal of the *Plano de Ações Estratégicas para o Enfrentamento das DCNT* (Strategic Action Plan to Tackle NCDs) in Brazil, 2011–2022, of reducing these rates by 2% per year. Such decline is consistent with the results of national and international studies, and may be assigned to initiatives in health, surveillance and comprehensive care promotion. The fight against smoking, the realization of the PNS, the monitoring by Vigitel and the redefinition of the Care for People with Chronic Diseases Network are noteworthy actions. The increased access to health services and the improvement of social indicators also seem to support the decreasing trend.

However, we observed that five capitals tend not to reach the goal: Boa Vista, Recife, São Luís, Campo Grande and Vitória. Among the factors that may explain this propensity are the health services overload (a relation better seen in São Luís and Boa Vista), low access to medicines for CRD in some regions, unfavorable social indicators and higher frequency of smoking (association more noted in Campo Grande). Nevertheless, it is possible to conjecture that there is influence of other factors in the time evolution of monthly mortality rates from CRD, especially for Vitória, capital with the highest average monthly household income *per capita* and schooling from six to 14 years of 100%.

Considering that CRD receive less investments in research and public attention, compared to other NCDs, this article contributes to a greater epidemiological knowledge of this group of diseases and to the monitoring of public health policies. We hope that this study allows a better planning of the public health system for the control of CRD and promotes initiatives in the research, surveillance and health care areas, especially in the capitals, and results show a trend not to achieve the goal of the Brazilian Ministry of Health.
