# Supplementary material for: Mortality from chronic respiratory disease in Brazil: time trend and forecasts
Source: Rev Saude Publica. 2022 Jun 7;56:52. doi: 10.11606/s1518-8787.2022056003672 (PMC9239334; doi:10.11606/s1518-8787.2022056003672)
Supplement: Translation

# Translation: Mortalidade por doença respiratória crônica no Brasil: tendência temporal e projeções

**Authors:** Marcio Sacramento de Oliveira, Elisa Hypólito Montovani, Maria de Fátima Ebole de Santana, Antonio Carlos Monteiro Ponce de Leon, Márcio Candeias Marques

**Affiliations:** I Brasil Fundação Oswaldo Cruz. Instituto de Comunicação e Informação Científica e Tecnológica em Saúde. Rio de Janeiro, RJ, Brasil; II Brasil Universidade do Estado do Rio de Janeiro. Instituto de Medicina Social. Rio de Janeiro, RJ, Brasil; III Brasil Fundação Oswaldo Cruz. Escola Politécnica de Saúde Joaquim Venâncio. Rio de Janeiro, RJ, Brasil

**Keywords:** Doenças Respiratórias, mortalidade, Indicadores de Doenças Crônicas, Planos e Programas de Saúde, tendências, Estudos Ecológicos

## Abstract

**OBJETIVO:**

Analisar a tendência temporal das taxas mensais de mortalidade por doenças respiratórias crônicas no Brasil de 1996 até 2017, com projeções para 2022, além de analisar a possibilidade de cumprimento da meta do Plano de Ações Estratégicas para o Enfrentamento das Doenças Crônicas Não Transmissíveis no Brasil de 2011 até 2022.

**MÉTODOS:**

Trata-se de estudo ecológico que utiliza dados do Sistema de Informações sobre Mortalidade, do Sistema de Informações Demográficas e Socioeconômicas e da Pesquisa Nacional por Amostra de Domicílios Contínua. O recorte etário foi estabelecido entre 30 e 69 anos e a evolução das taxas no tempo foi feita por meio de modelos autorregressivos integrados de média móvel em plataforma estatística R.

**RESULTADOS:**

As taxas de mortalidade precoce por doenças respiratórias crônicas apresentam-se decrescentes no Brasil como um todo e na maior parte das capitais, assim como, há tendência a atingir a meta do Ministério da Saúde na maior parte do país. Para capitais que tendem a não atingir a meta, verifica-se associação entre mortalidade e indicadores sociais, rede assistencial de saúde e frequência do tabagismo.

**CONCLUSÃO:**

Pretende-se que o estudo possibilite um melhor planejamento do sistema público de saúde para o controle das doenças respiratórias crônicas.

## INTRODUÇÃO

A taxa de mortalidade prematura global por doenças crônicas não transmissíveis (DCNT) sofreu redução de 18% no início deste século^1^. Entretanto, tal progresso não foi sustentado, visto que se observou desaceleração no declínio da taxa de mortalidade por DCNT^1^. Essas doenças, principalmente cardiovasculares, cânceres, doenças respiratórias crônicas e diabetes, correspondem à maior parcela de mortes no mundo (63% anualmente) e, uma vez que mudanças demográficas compensam a queda nas taxas de mortalidade, as DCNT estão assumindo uma proporção ainda maior do total de óbitos^1,2^.

Nas Américas, a taxa de redução percentual da mortalidade prematura por DCNT também diminuiu^3^. No Brasil, apesar de liderarem como causa de morbimortalidade, houve declínio de 20% na taxa de mortalidade por DCNT no período entre 1996 e 2007^4^, e redução na taxa de mortalidade prematura (30 a 69 anos) por DCNT, de 2000 a 2013 (2,5% ao ano), com liderança das doenças respiratórias crônicas (DRC) na variação percentual (4,1%)^5^.

Somente as DRC foram a terceira causa de morte (7%) no mundo em 2017, atrás de doenças cardiovasculares e neoplasias, com destaque para doença pulmonar obstrutiva crônica (DPOC) e asma^6^. Entre 1990 e 2017, o número total de mortes causadas por DRC aumentou mundialmente, enquanto a taxa de mortalidade padronizada por idade diminuiu^6,7^. O cenário das DRC no Brasil seguiu a tendência global, sendo a DPOC a quarta causa de óbitos em 2019, com aumento absoluto do número de mortes por DRC e redução das taxas de mortalidade quando ajustadas por idade^5,8^.

As DCNT afetam principalmente populações de baixa renda e escolaridade, pois estão mais expostas aos fatores de risco e têm menor acesso aos serviços de saúde^4,11^. No caso das DRC não é diferente. O tabagismo é o principal fator de risco associado a anos de vida ajustados por incapacidade^8^. Nesse contexto, estudos revelam que grupos menos favorecidos socioeconomicamente são mais propensos ao uso frequente do tabaco^12^. Somam-se a isso, dados da Vigilância de Fatores de Risco e Proteção para Doenças Crônicas por Inquérito Telefônico (Vigitel)^13^, que sinalizam diminuição da frequência do tabagismo com o aumento da escolaridade. Além disso, os mais pobres são menos diagnosticados com DRC e os que utilizam menos medicamentos^14^.

Diante do impacto das DCNT, políticas globais e nacionais firmaram o compromisso de enfrentamento dessas doenças. Destaques na agenda mundial foram: a primeira Reunião de Alto Nível das Nações Unidas sobre prevenção e controle de DCNT em 2011; a meta 3.4 do Objetivo de Desenvolvimento Sustentável (ODS), de reduzir em um terço a mortalidade prematura por essas doenças até 2030; e o Plano de Ação Global da Organização Mundial da Saúde para a Prevenção e Controle das DCNT 2013–2020^2,15^.

No Brasil, foi relevante a organização da Vigilância de DCNT, que permitiu estruturar ações para monitoramento de fatores de risco, morbidade e mortalidade das DCNT^4,16^. O Ministério da Saúde (MS) também construiu o Plano de Ações Estratégicas para o Enfrentamento das DCNT no Brasil, 2011–2022, tendo como uma das metas reduzir a taxa de mortalidade prematura por DCNT em 2% ao ano^4^.

O país tem sido considerado um exemplo no enfrentamento ao tabagismo, reconhecido por órgãos mundiais, com destaque para o aumento da taxação aos cigarros e o aumento de advertências nas embalagens, pela Lei nº 12.546 de 2011^16^.

Entretanto, globalmente, as DRC receberam menos recursos para pesquisa e menor atenção pública do que outras doenças, como as cardiovasculares e neoplasias^17^. Assim, o presente estudo pretende analisar a tendência temporal da mortalidade por DRC no Brasil, fazendo-se previsão dessas taxas para 2022, além de analisar, para cada capital, a possibilidade de cumprimento da meta do Plano de Ações Estratégicas para o Enfrentamento das DCNT no Brasil de 2011 a 2022.

## MÉTODOS

Trata-se de um estudo ecológico de séries temporais das taxas mensais de mortalidade por DRC (CID-10: J30-J98) no período entre 1996 e 2017 com previsão para 2022, nas capitais brasileiras e no Brasil como um todo. Os dados de óbito foram obtidos a partir do Sistema de Informações sobre Mortalidade do Datasus – Ministério da Saúde (SIM/MS), enquanto os dados populacionais dos anos de 1996 a 2015 foram adquiridos do Sistema de Informações Demográficas e Socioeconômicas, também do Datasus – MS. A estimativa do total populacional para 2016 e 2017 foi realizada a partir da Pesquisa Nacional por Amostra de Domicílios contínua, utilizando-se os pesos amostrais para o cálculo do total populacional.

Foram calculadas as taxas de mortalidade geral bruta mensais por DRC, e as taxas padronizadas por idade mensais, por meio do método de padronização direto, possibilitando a comparação dos resultados das diferentes populações por faixa etária. O método seleciona uma população padrão e aplica a taxa de mortalidade de todas as unidades para essa mesma população. Foi utilizada a população do Brasil em 2010, ano censitário. As taxas de mortalidade foram calculadas e representadas por 100.000 habitantes.

O recorte etário selecionado foi o de 30 a 69 anos, permitindo comparar a tendência temporal de mortalidade por DRC no Brasil e a meta proposta pelo Plano de Ações Estratégicas para o Enfrentamento das DCNT.

Para explorar o comportamento das taxas mensais de mortalidade por DRC, realizou-se análise descritiva dos dados, com obtenção de valores de mínimo, primeiro quartil, mediana, média, terceiro quartil, máximo e desvio padrão. Com base em média e desvio padrão, pode-se calcular o coeficiente de variação das taxas, importante para expressar a dispersão dos dados e compará-los sem influência das diferenças de magnitude da variável.

A evolução das taxas de mortalidade com relação ao tempo (meses/anos) foi feita por meio de modelos autorregressivos integrados de média móvel (ARIMA - do inglês *autoregressive integrated moving average*). Os modelos de séries temporais desse tipo permitem que a variável dependente, mortalidade por DRC, seja representado por Y_t_, explicado por valores defasados (Y_t_-_n_), do próprio Y_t_, diferente dos modelos de regressão, no qual Y_t_ é explicado pelos regressores µ, X1, X2, X3, … Xn, sem influência do tempo^18^. Assim, µ é a constante do modelo e X1, X2, X3, … Xn são variáveis explicativas. Para complementar os modelos ARIMA, foi utilizado também o modelo com defasamento distribuído autorregressivo (regressão dinâmica). As variáveis utilizadas foram a taxa de mortalidade padronizada, o tempo e a própria taxa de mortalidade defasada no tempo (t-1).

Assumindo que a taxa de mortalidade segue uma distribuição normal (gaussiana) e que é uma realização de um processo estocástico estacionário, aplicou-se a regressão dinâmica para cada capital, bem como para o Brasil. A ordem de defasagem para cada capital e Brasil (t-1) foi determinada pela função de autocorrelação. Sendo o modelo de forma geral dado por:


$$y_{t}\;\;=\;\alpha \;+\;\beta x_{t}\;\;+\;\gamma_{1}\;y_{t-1}+\;\gamma_{2}\;y_{t-2}+\;\gamma_{3}\;y_{t-3}+\;\theta_{1}\;u_{t-1}+\;\theta_{2}\;u_{t-2}+\;\theta_{3}\;u_{t-3}+\;\Theta_{1}\;u_{t-12}+\;\Gamma_{1}\;y_{t-12}+\;u_{t}$$

Onde *Y*_t_ é o valor da variável dependente no instante *t*; γ é o parâmetro ligado ao operador autorregressivo; θ é o parâmetro ligado ao operador de média móvel; e Θ e Γ são fatores sazonais. Para modelos ARIMA a primeira diferença foi denotada como $∆z_{t}\;\;=\;{z_{t}}_{\;}–\;z_{t-1}$ , sendo *Y*_*t*_ = ∆*z*_*t*_ para efeito da escrita dos modelos estimados. As componentes tendência, sazonalidade, ciclo e erro foram decompostas pela metodologia X12-ARIMA e a tendência avaliada como crescente ou decrescente. Foi realizado o teste Dickey-Fuller aumentado para todas as séries, que apresentaram raiz unitária, por essa, razão foi trabalhada a primeira diferença. Foi também observado um decaimento lento das funções de autocorrelação.

A estimação do modelo foi feita pela função de máxima verossimilhança. Para o processo de modelagem, suprimiram-se os três primeiros anos do Rio de Janeiro.

Já em relação a 19 capitais (Belém, Boa Vista, Macapá, Manaus, Palmas, Porto Velho, Rio Branco, João Pessoa, Maceió, Salvador, São Luís, Teresina, Brasília, Campo Grande, Cuiabá, Goiânia, Vitória, Curitiba e Florianópolis), foi necessário substituir os *outliers* pelas médias das taxas de mortalidade de seus respectivos anos para que se alcançasse melhor adequação dos modelos.

O desempenho geral do modelo foi avaliado a partir da previsão das taxas mensais de mortalidade por DRC para 2017 e da comparação aos valores reais desse ano, o que possibilitou calcular o percentual do erro médio absoluto das previsões (*mean absolute percentage error* – MAPE). Nesse procedimento, as taxas de 2017 foram suprimidas do modelo e estimou-se o modelo no restante. Definiu-se como 50% o valor máximo do MAPE para que o modelo fosse considerado válido para previsão. Após o modelo validado, foi realizada a previsão até dezembro de 2022.

Foi calculada a variação percentual prevista para a taxa de mortalidade com base no valor da taxa padronizada anual de 2011, ano de elaboração do plano do Ministério da Saúde, e na taxa de mortalidade prevista para 2022. O resultado de cada capital brasileira, bem como o de todo Brasil, foi comparado à proposta do documento (queda de 19,93% na taxa de mortalidade prematura até 2022, valor obtido a partir do cálculo da taxa acumulada de 2% ao ano), avaliando-se quais unidades demonstram tendência ou não a atingir a meta.

Os dados foram analisados em plataforma estatística R^19^, versão 3.6.3 utilizando os pacotes Forecast^20^ e Imtest^21^.

## RESULTADOS

A partir da análise de valores mínimos e máximos das taxas de mortalidade, apresentados na Tabela 1, as taxas mensais mais baixas foram observadas em Boa Vista, Palmas, Porto Velho, Rio Branco, Aracaju, João Pessoa, São Luís, Teresina, Campo Grande, Vitória e Florianópolis. Já o valor mais alto foi encontrado em Boa Vista (15,04%).

**Tabela 1:** Análise descritiva das taxas mensais de mortalidade por doenças respiratórias crônicas, por 100.000 habitantes, das capitais brasileiras e do Brasil como um todo, 1996–2017.

Local	Min.	Q1	Mediana	Média	Q3	Max.	DP
Brasil	1,32	1,88	2,19	2,33	2,72	4,59	0,59
Belém	0,60	2,02	2,72	3,05	3,82	8,38	1,44
Boa Vista	0,00	0,00	1,34	1,87	2,71	15,04	2,33
Macapá	0,26	0,99	1,50	1,59	2,02	5,32	0,78
Manaus	0,31	1,23	1,83	1,92	2,46	4,82	0,88
Palmas	0,00	0,00	0,93	1,74	2,85	20,09	2,60
Porto Velho	0,00	1,43	2,66	3,18	4,23	12,54	2,40
Rio Branco	0,00	1,67	3,22	3,40	4,87	12,71	2,28
Aracaju	0,00	1,08	1,92	2,33	3,42	7,83	1,56
Fortaleza	0,44	1,46	2,10	2,33	3,04	5,66	1,06
João Pessoa	0,00	1,32	1,92	2,14	2,80	7,29	1,20
Maceió	0,35	1,61	2,45	2,78	3,64	8,61	1,50
Natal	0,00	0,79	1,21	1,33	1,71	4,25	0,80
Recife	1,21	2,34	2,88	2,99	3,57	6,22	0,90
Salvador	0,72	2,24	3,41	3,54	4,63	8,94	1,56
São Luís	0,00	1,19	1,77	1,97	2,50	5,72	1,08
Teresina	0,00	1,07	1,70	1,88	2,50	7,08	1,14
Brasília	0,26	0,99	1,50	1,59	2,02	5,32	0,78
Campo Grande	0,00	1,33	2,02	2,10	2,74	6,12	1,05
Cuiabá	0,00	1,44	2,38	2,59	3,39	9,43	1,55
Goiânia	0,58	1,79	2,46	2,61	3,19	6,63	1,14
Belo Horizonte	0,52	1,18	1,68	1,92	2,46	6,25	0,97
Rio de Janeiro	1,20	1,83	2,24	2,42	2,88	5,15	0,78
São Paulo	1,13	1,70	2,12	2,25	2,73	5,07	0,70
Vitória	0,00	0,56	1,14	1,29	1,90	3,94	0,97
Curitiba	0,35	1,26	1,93	2,23	2,93	7,88	1,31
Florianópolis	0,00	0,93	1,62	1,92	2,59	7,09	1,32
Porto Alegre	0,93	2,08	2,73	3,06	3,88	9,90	1,43

Q1: primeiro quartil ; Q3: terceiro quartil; DP: desvio padrão.

A comparação entre os dados de média e mediana da Tabela 1, para as taxas de mortalidade de cada capital, demonstraram que Palmas tem o maior número de *outliers*.

Foi observada variação significativa nas taxas mensais de mortalidade por DRC em todas as unidades estudadas, o que pode ser notado a partir dos seus valores de média e desvio padrão apresentados na Tabela 1. Todas as capitais, assim como o Brasil como um todo, apresentaram coeficiente de variação superior a 25%, sendo o menor valor calculado para o país (25,14%). Por sua vez, Boa Vista e Palmas revelaram coeficientes de variação superiores a 100% (respectivamente, 124,41% e 149,49%).

Sobre a precisão das previsões, observa-se no MAPE, na Tabela 2, que os modelos do Brasil (2,94%), São Paulo (12,93%) e Rio de Janeiro (13,81%) possuem o menor erro percentual médio em relação aos demais. Já os modelos de Boa Vista, Macapá, Manaus, Aracaju, João Pessoa, Campo Grande e Vitória apresentaram altos valores de erro, revelando uma capacidade de previsão pouco precisa. Já as capitais Porto Velho, Rio Branco, Natal, Teresina e Cuiabá, os valores do erro traduzem que seus modelos não são adequados para realizar previsão. Cuiabá, além do erro percentual médio alto, revelou um modelo com comportamento em níveis, tornando difícil a análise da tendência.

**Tabela 2:** Modelo preditivo, desempenho e tendência das taxas mensais de mortalidade por doenças respiratórias crônicas, por 100.000 habitantes, das capitais brasileiras e do Brasil como um todo, 1996–2017.

Local	Equação	Sigma2	MAPE (%)	Tendência
Brasil	∆*ẑ*_*t*_ = 0,5785∆*z*_*t*–1_ – 0,9253*u*_*t*–1_ – 0,4879*u*_*t*–12_ –0,2251*u*_*t*–13_	0,02	2,94	Decrescente
Belém	∆*ẑ*_*t*_ = –0,4177∆*z*_*t*–1_ – 0,3695*u*_*t*–1_ – 0,4865*u*_*t*–2_ – 0,8696∆*z*_*t*–12_ + 0,0281*u*_*t*–1*2*_ – 0,7212*u*_*t*–13_ – 0,0964*u*_*t*–14_ – 0,2105*u*_*t*–15_	1,06	29,16	Crescente
Boa Vista	∆*ẑ*_*t*_ = –0,8365∆*z*_*t*–1_ – 0,1131*u*_*t*–1_ – 0,8126*u*_*t*–2_ + 0,2129∆*z*_*t*–12_ – 0,1860*u*_*t*–12_ – 0,0713*u*_*t*–2_	2,34	39,13	Decrescente
Macapá	∆*ẑ*_*t*_ = –0,1152∆*z*_*t*–1_ –0,9375*u*_*t*–1_ –0,610∆*z*_*t*–12_ + 0,6822*u*_*t*–12_ + 0,1924*u*_*t*–13_ –0,0045*x*_*t*_	1,79	41,33	Decrescente
Manaus	∆*ẑ*_*t*_ = –0,0755∆*z*_*t*–1_–0,9305*u*_*t*–1_ + 0,8866∆*z*_*t*–12_ –0,8506*u*_*t*–12_ + 0,1254*u*_*t*–13_ –0,0042*x*_*t*_	0,58	44,37	Decrescente
Palmas	∆*ẑ*_*t*_ = –0,6530∆*z*_*t*–1_ – 0,2855*u*_*t*–1_ – 0,6947*u*_*t*–2_ + 0,1092*u*_*t*–3_ – 0,1290*u*_*t*–4_ – 0,4751∆*z*_*t*–12_	3,69	29,42	Crescente
Porto Velho	∆*ẑ*_*t*_ = –0,8657*u*_*t*–1_–0,1249*u*_*t*–12_	2,86	69,79	NA
Rio Branco	∆*ẑ*_*t*_ = –0,7347∆*z*_*t*–1_ – 0,5022∆*z*_*t*–2_ – 0,3746∆*z*_*t*–3_ – 0,2346∆*z*_*t*–4_	5,23	83,79	NA
Aracaju	∆*ẑ*_*t*_ = –0,0973∆*z*_*t*–1_ + 0,0956∆*z*_*t*–2_ – 0,2061∆*z*_*t*–3_ – 0,8573*u*_*t*–1_	1,52	40,99	Decrescente
Fortaleza	∆*ẑ*_*t*_ = 0,2635∆*z*_*t*–1_ – 0,9302*u*_*t*–1_ – 0,0085*x*_*t*_	0,48	28,87	Decrescente
João Pessoa	∆*ẑ*_*t*_ = –0,7849∆*z*_*t*–1_ – 0,0480*u*_*t*–1_ – 0,7135*u*_*t*–2_ – 0,0083*x*_*t*_	0,92	49,62	Decrescente
Maceió	∆*ẑ*_*t*_ = –0,8756∆*z*_*t*–1_ + 0,0225∆*z*_*t*–12_ – 0,0163*u*_*t*–12_ – 0,0104*x*_*t*_	1,16	34,27	Decrescente
Natal	∆*ẑ*_*t*_ = –0,8459*u*_*t*–1_	0,42	91,66	NA
Recife	∆*ẑ*_*t*_ = –0,8636*u*_*t*–1_ – 0,7568∆*z*_*t*–12_ – 0,0654∆*z*_*t*–13_ + 0,7071*u*_*t*–12_	0,76	22,81	Crescente
Salvador	∆*ẑ*_*t*_ = 0,2305∆*z*_*t*–1_ – 0,9429*u*_*t*–1_ – 0,0161*x*_*t*_	0,60	28,55	Decrescente
São Luís	∆*ẑ*_*t*_ = 0,1169∆*z*_*t*–1_ – 0,6843∆*z*_*t*–2_ – 0,9558*u*_*t*–1_ + 0,5828*u*_*t*–2_ – 0,4866*u*_*t*–3_ – 0,0225*u*_*t*–4_ – 0,0824*u*_*t*–5_	0,63	35,33	Crescente
Teresina	∆*ẑ*_*t*_ = 0,1373∆*z*_*t*–1_ – 0,9492*u*_*t*–1_ + 0,1342∆*z*_*t*–12_ – 0,0171∆*z*_*t*–13_	0,70	104,75	NA
Brasília	∆*ẑ*_*t*_ = –0,1638∆*z*_*t*–1_ – 0,8374∆*z*_*t*–2_ – 0,0663∆*z*_*t*–3_ – 0,4886∆*z*_*t*–4_ – 0,6258*u*_*t*–1_ + 0,9044*u*_*t*–2_ – 0,9111*u*_*t*–3_ + 0,4809*u*_*t*–4_ – 0,7722*u*_*t*–5_ + 0,0348∆*z*_*t*–12_ + 0,1801∆*z*_*t*–13_ – 0,0076*x*_*t*_	0,27	41,60	Decrescente
Campo Grande	∆*ẑ*_*t*_ = –0,7989∆*z*_*t*–1_ + 0,1317∆*z*_*t*–2_ + 0,1151∆*z*_*t*–3_–0,1016*u*_*t*–1_–0,8287*u*_*t*–2_ + 0,0206∆*z*_*t*–12_ + 0,0883∆*z*_*t*–13_	0,78	33,33	Crescente
Cuiabá	∆*ẑ*_*t*_ = –0,9197*u*_*t*–1_	1,40	162,21	NA
Goiânia	∆*ẑ*_*t*_ = 0,0376∆*z*_*t*–1_ + 0,1849∆*z*_*t*–2_ – 0,8867*u*_*t*–1_ – 0,6050∆*z*_*t*–12_ – 0,2614∆*z*_*t*–13_	0,86	33,58	Decrescente
Belo Horizonte	∆*ẑ*_*t*_ = –0,6022∆*z*_*t*–1_ – 0,8742∆*z*_*t*–2_ – 0,2019*u*_*t*–1_ + 0,3535*u*_*t*–2_ – 0,7581*u*_*t*–3_ – 0,2362*u*_*t*–4_ + 0,2448∆*z*_*t*–12_ – 0,010*x*_*t*_	0,28	25,10	Decrescente
Rio de Janeiro	∆*ẑ*_*t*_ = 0,3222∆*z*_*t*–1_ + 0,1314∆*z*_*t*–2_ – 0,9127*u*_*t*–1_ + 0,1836∆*z*_*t*–12_ – 0,0110*x*_*t*_	0,15	13,81	Decrescente
São Paulo	∆*ẑ*_*t*_ = 0,3580∆*z*_*t*–1_ – 0,9188*u*_*t*–1_ + 0,2552*u*_*t*–12_ + 0,2628*u*_*t*–13_ – 0,0071*x*_*t*_	0,12	12,93	Decrescente
Vitória	∆*ẑ*_*t*_ = –0,7866∆*z*_*t*–1_ – 0,1029∆*z*_*t*–2_ – 0,1812*u*_*t*–1_ – 0,7338*u*_*t*–2_ + 0,4307∆*z*_*t*–12_ – 0,4943*u*_*t*–12_	0,91	47,10	Crescente
Curitiba	∆*ẑ*_*t*_ = 0,02542∆*z*_*t*–1_ – 0,9633*u*_*t*–1_ – 0,7916∆*z*_*t*–12_ + 0,139*u*_*t*–12_ – 0,5454*u*_*t*–13_	0,52	35,63	Crescente
Florianópolis	∆*ẑ*_*t*_ = –0,9353*u*_*t*–1_ – 0,8766*u*_*t*–12_	1,10	37,01	Decrescente
Porto Alegre	∆*ẑ*_*t*_ = 0,0204∆*z*_*t*–1_ – 0,7605∆*z*_*t*–2_ – 0,6562*u*_*t*–1_ + 0,6890*u*_*t*–2_ – 0,7344*u*_*t*–3_ – 0,1944*u*_*t*–4_ + 0,3525∆*z*_*t*–12_ + 0,2395∆*z*_*t*–13_	0,80	34,00	Decrescente

NA: não se aplica; MAPE: do inglês *mean absolute percentage error*.

Com base na Tabela 2 e nas Figuras 1 e 2, nota-se que as taxas de mortalidade precoce por DRC apresentam-se decrescentes no Brasil e em 15 das 22 capitais que tiveram os modelos validados. Belém, Palmas, Recife, São Luís, Campo Grande, Vitória e Curitiba, por outro lado, mostram essas taxas em uma tendência crescente. Para o Rio de Janeiro, os dados de mortalidade por DRC nos anos entre 1996 e 1998 foram muito abaixo dos demais, indicando uma provável subnotificação nesse período e, portanto, foram desconsiderados.

**Figura 1:** Taxas mensais de mortalidade por doenças respiratórias crônicas ajustadas para Brasil como um todo, Belém, Boa Vista, Macapá, Manaus, Palmas, Aracaju, Fortaleza, João Pessoa, Maceió, Recife e Salvador – de 1996 a 2016, com 2017 para validação e previsão das taxas mensais de mortalidade por DRC de 01/01/2017 a 12/12/2022 – Previsão a partir de um modelo ARIMA.

DRC: doenças respiratórias crônicas; ARIMA: do inglês *autoregressive integrated moving average*.

**Figura 2:** Taxas mensais de mortalidade por doenças respiratórias crônicas ajustadas para Brasília, Campo Grande, Goiânia, Belo Horizonte, Rio de Janeiro, São Paulo, Vitória, Curitiba, Florianópolis e Porto Alegre – de 1996 a 2016, com 2017 para validação e previsão das taxas mensais de mortalidade por DRC de 01/01/2017 a 12/12/2022 – Previsão a partir de um modelo ARIMA.

DRC: doenças respiratórias crônicas; ARIMA: do inglês *autoregressive integrated moving average*.

Também se verifica, segundo a Tabela 3, que o Brasil como um todo e 16 das 22 capitais tendem a atingir a meta do Plano de Ações Estratégicas para o Enfrentamento das DCNT, pois apresentam variação percentual prevista para as taxas de mortalidade precoce por DRC superior àquela esperada (19,92%). Já para Boa Vista, Recife, São Luís, Campo Grande e Vitória, os resultados revelam tendência a não atingir a meta. A capital Boa Vista revelou a menor taxa em 2011 (10,04), enquanto a maior foi vista em Rio Branco (36,76).

**Tabela 3:** Taxas de mortalidade por doenças respiratórias crônicas, por 100.000 habitantes, previstas para 2022 e avaliação do cumprimento da meta do Plano de Ações Estratégicas para o Enfrentamento das DCNT, para Brasil e capitais brasileiras.

Local	Taxa padronizada 2011	Taxa padronizada 2022 (Plano Nacional)	Taxa padronizada 2022 (prevista)	Variação % prevista (2011–2022)	Meta do Plano Nacional
Brasil	23,65	18,94	17,45	-26,22	Tende a atingir
Belém	23,11	18,51	14,28	-38,19	Tende a atingir
Boa Vista	10,04	8,04	13,63	+35,80	Tende a não atingir
Macapá	14,85	11,89	11,03	-25,69	Tende a atingir
Manaus	20,51	16,42	10,69	-47,86	Tende a atingir
Palmas	18,69	14,97	10,28	-44,98	Tende a atingir
Porto Velho	30,51	24,43	NA	NA	NA
Rio Branco	36,76	29,43	NA	NA	NA
Aracaju	18,40	14,74	7,94	-56,87	Tende a atingir
Fortaleza	19,23	15,40	7,28	-62,14	Tende a atingir
João Pessoa	21,74	17,41	6,22	-71,39	Tende a atingir
Maceió	28,71	22,99	4,78	-83,35	Tende a atingir
Natal	13,35	10,69	NA	NA	NA
Recife	35,64	28,54	32,43	-9,01	Tende a não atingir
Salvador	33,30	26,66	4,86	-84,41	Tende a atingir
São Luís	16,28	13,04	17,01	+4,50	Tende a não atingir
Teresina	20,37	16,31	NA	NA	NA
Brasília	13,94	11,16	3,11	-77,70	Tende a atingir
Campo Grande	19,92	15,95	18,11	-9,13	Tende a não atingir
Cuiabá	21,84	17,49	NA	NA	NA
Goiânia	22,96	18,38	8,30	-63,84	Tende a atingir
Belo Horizonte	15,72	12,59	2,72	-82,67	Tende a atingir
Rio de Janeiro	24,58	19,68	8,94	-63,61	Tende a atingir
São Paulo	22,47	17,99	11,55	-48,62	Tende a atingir
Vitória	15,11	12,10	12,41	-17,86	Tende a não atingir
Curitiba	16,57	13,27	7,79	-52,97	Tende a atingir
Florianópolis	17,56	14,06	6,28	-64,20	Tende a atingir
Porto Alegre	30,89	24,73	23,25	-24,73	Tende a atingir

DCNT: doenças crônicas não transmissíveis; NA: não se aplica.

## DISCUSSÃO

A tendência de queda nas taxas de mortalidade precoce por DRC na maior parte do Brasil coincide com resultados de pesquisas globais, que revelam as maiores reduções na carga de DRC na América Latina, e de outros estudos brasileiros que apontam diminuição nessas taxas nas cinco regiões do país^5,6,8^. Esse declínio tem sido atribuído, entre outros fatores, aos avanços no combate ao tabagismo^5^. Ações nesse sentido estão relacionadas ao Eixo II – Promoção da Saúde – do Plano de Ações Estratégicas para o enfrentamento das DCNT no Brasil^4^. Também parece corroborar o aumento de acesso e uso de serviços de saúde, segundo estudo que avaliou dados da Pesquisa Nacional de Saúde (PNS) de 2013^22^.

Segundo a Síntese de Indicadores Sociais, do IBGE, houve queda na proporção de pessoas vivendo abaixo da linha da pobreza entre 2012 e 2014, um determinante social importante no processo saúde-doença, porém voltou a crescer a partir de 2015^23^. Outro determinante significativo é a escolaridade, que apresentou aumento no nível de instrução ao longo das últimas décadas pode ser relacionado com a redução da exposição aos fatores de risco para DRC, apesar de essa maior escolarização ter ocorrido de forma desigual entre as diferentes classes socioeconômicas^23^. Nesse contexto, parece haver relação entre mortalidade por asma e fatores como escolaridade e distribuição de renda, explicando a baixa mortalidade por essa doença em países de alta renda^24^. Já para DPOC, essa associação é menos monotônica e decorre de aumento da poluição do ar e tabagismo nos países em transição demográfica e epidemiológica, causalidade que merece investigação específica em estudo futuro^24^.

Sabendo-se que as maiores taxas de mortalidade por DRC foram encontradas no Sul da Ásia, um estudo que analisou a tendência de prevalência, letalidade e fatores de risco para DRC na Índia permite comparar a evolução do enfrentamento dessas doenças crônicas em outro país em desenvolvimento com a situação do Brasil^6,25^. A Política Nacional de Saúde da Índia de 2017 recomenda a redução da mortalidade por DCNT, inclusive por DRC, em 25% até 2025, enquanto o estudo revelou quedas maiores que 30% nas taxas brutas de letalidade para DPOC e asma entre 1990 e 2016^25^.

A tendência a atingir a meta na maior parte do Brasil está de acordo com estudo de Malta (2016), que descreveu os avanços do plano do MS após cinco anos de implementação, em que o monitoramento pelo Vigitel demonstrou redução na mortalidade prematura pelos quatro grupos principais de DCNT e diminuição do consumo de tabaco^26^. A pesquisa destaca outras ações em vigilância como a realização da PNS em 2013 e da Pesquisa Nacional de Saúde do Escolar desde 2009; além de iniciativas em promoção da saúde e cuidado integral, como a redefinição da Rede de Atenção à Pessoa com Doença Crônica, o lançamento do Programa Nacional de Melhoria do Acesso e da Qualidade e do Programa Farmácia Popular do Brasil^26^. Também se destacam as parcerias com instituições de ensino e pesquisa na estruturação de vigilância das DCNT e financiamento em 2011 para a implantação do plano do MS^16^.

Para melhor interpretar os resultados de tendência a atingir ou não atingir a meta, alguns fatores foram investigados: a situação da assistência em saúde; os indicadores sociais; e a exposição ao tabagismo.

A situação da assistência em saúde foi estudada por meio do Cadastro Nacional de Estabelecimentos de Saúde e de artigo que estimou o acesso ao tratamento medicamentoso de DRC^14,26^. São Luís e Boa Vista, capitais que tendem a não atingir a meta, apresentam elevada relação entre número populacional e número de estabelecimentos de saúde, a primeira com 1.056,15 habitantes para cada estabelecimento e a segunda com 1.055,17 pessoas por unidade, o que demonstra possível sobrecarga do sistema de saúde nesses municípios^26^. Entretanto, considerando apenas estabelecimentos de esfera jurídica pública, a razão entre população e número de estabelecimentos coloca Boa Vista como a capital com a segunda melhor relação, revelando menor sobrecarga do sistema público de saúde. Assim, ainda que essa razão seja alta, de 3.485,26 habitantes por estabelecimento público, sozinha não justifica a tendência do município de não atingir a meta do plano do MS, demonstrando provável influência de outros fatores.

Outra capital que tende a não atingir a meta é Recife, que tem uma relação de 720,19 habitantes por estabelecimento de saúde, a 12ª pior razão, melhorando seis posições quando feita a restrição às unidades públicas. Já Vitória e Campo Grande apresentam piores colocações em sobrecarga quando desconsiderados os estabelecimentos de esfera jurídica privada, a primeira apresentando a melhor relação entre todas as capitais quando considerados estabelecimentos públicos e privados. Portanto, a esfera privada seria a responsável pela redução da sobrecarga do sistema de saúde nessas capitais, que possivelmente não conseguiriam resolver a questão das DRC, uma vez que a maior parte das pessoas dependem do SUS. Desse modo, é possível que haja relação da razão entre população e número de estabelecimentos de saúde com a tendência temporal da mortalidade por DRC, o que é mais bem visto para São Luís e Boa Vista, mas essa influência não é clara e não pode ser analisada sem a consideração de outros fatores.

Sobre o acesso aos medicamentos para DRC, Leal et al.^14^ (2018) observou que, para indivíduos que afirmavam ter indicação médica de tratamento farmacológico, a prevalência do uso de medicamentos foi menor entre os residentes da região Norte (84%), seguida do Nordeste (92,8%)^14^. Como duas das cinco capitais que tendem a não atingir a meta de redução pertencem à região Nordeste, e uma à Norte, pode haver relação entre essa tendência e o baixo acesso ao tratamento medicamentoso, o que não é válido para Campo Grande, pois a região Centro-Oeste obteve a melhor prevalência (98%) de acesso aos medicamentos de DRC. Já a região Sudeste revelou prevalência de 89,7%, ficando em terceiro lugar^14^.

Quanto aos indicadores sociais, por um lado, São Luís é a segunda capital com menor rendimento médio mensal domiciliar *per capita* (R$ 1.043,00), maior apenas que o rendimento do Macapá. Por outro, Vitória, que também tende a não atingir a meta, tem o maior rendimento médio (R$ 2.988,00) entre todas as capitais^22^. Sobre concentração de renda, observou-se, a partir do Índice de Gini, índice clássico de desigualdade que revela a distribuição do rendimento domiciliar *per capita*, que Recife se destaca pela maior desigualdade na distribuição de renda^27^.

São Luís e Recife, assim como Boa Vista, também apresentam elevada proporção de pessoas abaixo da linha da pobreza, o que corrobora a ideia de que há influência dos indicadores sociais para a tendência da mortalidade por DRC nessas capitais^23^. Soma-se ainda a questão da escolarização, índice em que Recife e São Luís revelaram taxas inferiores à maior parte das capitais (98,80% e 98,50% respectivamente)^28^ entre pessoas com idades entre seis e 14 anos. De modo contrário, Vitória e Campo Grande mostraram escolarização dessa faixa etária de 100%^28^.

A frequência de adultos que fumam é maior em Porto Alegre (14,40%) e menor em São Luís (4,80%)^13^, Campo Grande também apresenta taxa elevada em relação às demais capitais (10,80%), enquanto a frequência em Boa Vista representa a mediana (7,20%), mesmo valor observado em Recife e próximo ao de Vitória (7,60%)^13^. A distribuição é parecida para o percentual de adultos que fumam 20 ou mais cigarros por dia, com liderança de Porto Alegre, sendo a frequência de São Luís superior apenas à de Macapá^13^. Logo, a associação entre a frequência desse fator de risco e a mortalidade por DRC é mais bem vista em Campo Grande.

A escolha do SIM como fonte dos dados de óbito baseou-se em estudos que revelam seu desempenho adequado, sendo bem avaliado em usabilidade, confiabilidade, segurança e qualidade em uso^29^. O SIM é o sistema de informação mais antigo do MS, implantado em 1976 e, desde então, amplia sua estabilidade e cobertura geográfica^29,30^. Contudo, estudos apontam limitações, sobretudo em interoperabilidade e, especialmente nas regiões Norte e Nordeste, elevados sub-registro e número de óbitos com causas não definidas^29,30^. Outra limitação do estudo está na geração de grandes intervalos de confiança dos modelos ARIMA, para a previsão para o ano de 2022, mesmo apresentando uma razoável capacidade preditiva com um erro percentual médio de 32,21% para 2017, ano deixado de fora da estimativa para fins de teste das previsões.

## CONCLUSÕES

O estudo demonstra que as taxas de mortalidade precoce por DRC apresentam-se decrescentes no Brasil como um todo e na maioria das capitais, bem como é observada na maior parte do país a tendência de atingir a meta do Plano de Ações Estratégicas para o Enfrentamento das DCNT no Brasil, 2011–2022, de redução dessas taxas em 2% ao ano. Verificou-se que tal declínio é coerente com resultados de pesquisas nacionais e internacionais, e pode ser atribuído a iniciativas nos eixos de promoção da saúde, vigilância e cuidado integral. Destacam-se o enfrentamento ao tabagismo, a realização da PNS, o monitoramento pelo Vigitel e a redefinição da Rede de Atenção à Pessoa com Doença Crônica. O aumento do acesso aos serviços de saúde e a melhoria dos indicadores sociais também parecem corroborar para a tendência de decrescimento.

Entretanto, observou-se que cinco capitais tendem a não atingir a meta: Boa Vista, Recife, São Luís, Campo Grande e Vitória. Entre os fatores que podem explicar tal propensão, estão a sobrecarga dos serviços de saúde (relação mais bem vista para São Luís e Boa Vista), baixo acesso aos medicamentos para DRC em algumas regiões, indicadores sociais desfavoráveis e maior frequência do tabagismo (associação mais notada para Campo Grande). Ainda assim, é possível conjecturar que há influência de outros fatores na evolução temporal das taxas mensais de mortalidade por DRC, especialmente para Vitória, capital com o maior rendimento médio mensal domiciliar *per capita* e escolarização de seis a 14 anos de 100%.

Considerando que as DRC recebem menores investimentos em pesquisa e atenção pública, comparada a outras DCNT, o presente artigo contribui para um maior conhecimento epidemiológico desse grupo de doenças e para o monitoramento das políticas públicas de saúde. Aspira-se que o estudo possibilite um melhor planejamento do sistema público de saúde para o controle das DRC e fomente iniciativas dos setores de pesquisa, vigilância e atenção em saúde, em especial nas capitais cujos resultados mostram tendência a não cumprir a meta do Ministério da Saúde.
